# Restrictive pulmonary function is more prevalent in patients with ankylosing spondylitis than in matched population controls and is associated with impaired spinal mobility: a comparative study

**DOI:** 10.1186/ar3699

**Published:** 2012-01-25

**Authors:** Gunnhild Berdal, Silje Halvorsen, Désirée van der Heijde, Morten Mowe, Hanne Dagfinrud

**Affiliations:** 1 National Resource Center for Rehabilitation in Rheumatology, Diakonhjemmet Hospital, PO Box 23 Vinderen, No-0319 Oslo, Norway; 2 Department of Health Sciences, Faculty of Medicine, University of Oslo, PO Box 1089 Blindern, No-0318 Oslo, Norway; 3 Oslo University Hospital, Department of Physiotherapy, Medical Clinic, PO Box 4956 Nydalen, No-0424 Oslo, Norway; 4 Department of Rheumatology, Leiden University Medical Center, PO Box 9600, 2300 RC Leiden, the Netherlands; 5 Oslo University Hospital, Department of General Internal Medicine, PO Box 4959 Nydalen, No-0424 Oslo, Norway; 6 Institute of Clinical Medicine, Faculty of Medicine, University of Oslo, PO Box 4959 Nydalen, No-0424 Oslo, Norway

## Abstract

**Introduction:**

Pulmonary involvement is a known manifestation in patients with ankylosing spondylitis (AS). However, previous studies have been based on small samples and the reported prevalence and associations with typical clinical features vary. The purpose of this study was to compare pulmonary function (PF) in patients with AS and population controls, and to study associations between PF and disease related variables, cardio-respiratory fitness and demographic variables in patients with AS.

**Methods:**

In a cross-sectional controlled study, 147 AS patients and 121 controls underwent examinations, including demographic variables, laboratory (C-reactive protein (CRP), erythrocyte sedimentation rate (ESR)) and clinical measures (disease activity (AS disease activity score, ASDAS), physical function (Bath ankylosing spondylitis functional index, BASFI), spinal mobility (Bath ankylosing spondylitis metrology index, BASMI), chest expansion, cardio-respiratory fitness (peak oxygen uptake, VO_2_peak) and pulmonary function test (PFT) (spirometry)). Cumulative probability plots were used to visualize associations between the ASDAS and BASMI scores and the corresponding forced vital capacity (FVC%, percentage of predicted value controlled for the influence of confounding factors) score for each patient. Univariate ANCOVAs were performed to explore group differences in PF adjusting for relevant variables, and a multiple regression model was used to estimate the explanatory power of independent variables (demographic, disease related, VO_2_peak) on restrictive ventilatory impairment (FVC%).

**Results:**

AS patients showed significantly lower PF values compared with controls, and significantly more patients were categorized with restrictive pattern (18% vs. 0%, *P *< 0.001). Cumulative probability plots showed significant associations between spinal mobility measures (BASMI) and FVC% for individual patients. BASMI, chest expansion and male gender contributed significantly and independently in a multiple regression model predicting the variation of FVC% in AS patients, whereas disease activity, physical function and VO_2peak _did not contribute significantly. The final model explained 45% of the variance in FVC% (*P *< 0.001).

**Conclusions:**

This study showed significantly impaired pulmonary function in the AS patients compared to controls and reference data, and demonstrated a clear relationship between reduced spinal mobility and restrictive PF in AS patients. The results support the assumption of an association between musculoskeletal limitations and restrictive respiratory impairment in AS, emphasizing the importance of maintaining spinal flexibility in the management of the disease. Further, patients with severely reduced spinal mobility should be referred for pulmonary function examination and relevant follow-up treatment.

## Introduction

Ankylosing spondylitis is a chronic, systemic, inflammatory, rheumatic disease affecting mainly the axial skeleton and sacroiliac joints, causing characteristic inflammatory back pain and resulting in varying degree of structural and functional impairments [1]. AS may also be associated with extra-spinal manifestations, involving peripheral joints, eye, skin, bowel and an increased risk of cardiovascular morbidity [2]. Additionally, pulmonary involvement is a known manifestation, emerging either as interstitial lung disease or as a consequence of chest wall abnormalities [3,4]. Both of these conditions may lead to restrictive pulmonary function, typically presented as restrictive pattern in a pulmonary function test (spirometry).

AS is characterized by inflammation in the thoracic vertebrae and in the costovertebral joints, causing gradual fusion and ossification of the joints, for some patients ultimately resulting in increased dorsal kyphosis, rigidity of the thorax and permanent chest wall immobility [5,6]. Reduced lung volumes have been suggested to be a consequence of mechanical limitations, due to bony ankylosis of the thoracic joints [7], because restrictive respiratory impairment frequently has been reported to be associated with low thoracic expansibility [6-10]. Additionally, some claim that ongoing inflammatory processes in the thoracic joints may explain limitations of chest excursions, by causing pain and stiffness, and thus contributing to reduced pulmonary function [5,9,11]. Others suggest that pleuropulmonary tissue is an independent primary target in AS [12,13], and that inflammatory processes in the lung parenchyma with a tendency to fibrosis might be as significant as mechanical factors in the development of reduced pulmonary function in AS [9,14,15]. However, reports have differed regarding whether pulmonary function worsens with disease progression [5], and whether respiratory restriction correlates with limitation of chest wall movements [5,6,13,16,17].

Further, the frequency of pulmonary involvement varies depending on the diagnostic method applied, and has been reported to be between 20 and 57% using spirometry [4,8,12,18], between 1 and 15% with radiographic evaluation, and between 40 and 80% in studies in which high resolution computed tomography was applied [4,9,13,14,19-21].

Previous studies on this field have been based on small study samples. Hence, the associations between anthropometric, musculoskeletal and disease related factors and pulmonary function abnormalities in AS patients need to be more thoroughly explored.

The aims of this study were to characterize pulmonary function variables in patients with AS, and to examine whether, and in what respect, these variables differ from those observed in population controls. Also we aimed to investigate possible associations between pulmonary function and demographic, disease specific and laboratory measures and cardio-respiratory fitness.

This article provides findings from a comprehensive clinical assessment of patients with AS, and the results are compared to population controls.

## Materials and methods

### Study design and selection of patients and population controls

In a cross-sectional study, patients clinically diagnosed with AS by a rheumatologist and aged between 18 and 70 years were recruited from a hospital-based register. Additionally, 121 population controls were randomly selected from the national register by Statistics Norway to match age, gender and residential area of the AS patients. The only exclusion criterion was a history of inflammatory arthritis.

### Ethics

All participants gave their written consent before inclusion, and the procedures followed The World Medical Association Declaration of Helsinki. The protocol was reviewed and approved by The National Committee for Medical Research Ethics, Southern Norway (S-02059 and S-03066), and the Norwegian Data Inspectorate provided licence to store and register individual health information (08/00165-2/sve).

### Clinical assessments and self-reported data

Demographic variables (age, disease duration, education level, smoking history, work and marital status) were recorded for all participants using a questionnaire. Disease activity was measured by inflammatory markers (C-reactive protein (mg/l), CRP), erythrocyte sedimentation rate (mm/h), ESR) and by the AS Disease Activity Score (ASDAS) [22]. The ASDAS includes CRP-levels (mg/l) in addition to patient assessment of peripheral joint pain/swelling, total back pain, duration of morning stiffness (Bath Ankylosing Spondylitis Disease Activity Index, BASDAI, Q1, Q2 and Q6 [23]) and patient global assessment of disease activity (0 to 10). The scores were categorized according to published cut-offs with low ASDAS defined as < 1.3, moderate ASDAS < 2.1, high ASDAS ≤ 3.5 and very high ASDAS > 3.5 [24]. The ASDAS can be used to discriminate between groups of patients, and it provides information about the actual disease activity state that has been reached [22].

Patient-reported physical function was measured with the Bath Ankylosing Spondylitis Functional Index (BASFI). BASFI consists of eight questions relating to specific functions on activity level and two questions reflecting the person's ability to cope with everyday life. The responses were given on NRS. The mean score of 10 items gave the final BASFI score ranging from 0 (easy) to 10 (impossible) [25].

Anthropometric measures were examined by the Bath Ankylosing Spondylitis Metrology Index (BASMI) [26], by chest expansion and by body mass index (BMI). BASMI includes five clinical examinations of the spinal column and the hip joints; that is the distance from tragus to wall (TWD), lumbar flexion (l-Schober), lateral lumbar flexion, cervical rotation and inter-malleolar distance. Each of the five measurements was classified into 11 equal sections, and the mean of the five scores produced a BASMI score from 0 to 10; low score indicating normal function. BASMI is suitable for assessing spinal mobility across the whole range of disease severity [27]. The test is comprehensive, quick, reproducible and sensitive to change across the disease spectrum [26], and is shown to be valid, reliable and responsive [28,29]. Chest expansion was measured with tape measure placed circumferentially around the chest on level with the xiphoid process. The difference (cm) in circumference of the chest between maximum inspiration and maximum expiration was recorded (best of two attempts, rounded at 0.1 cm) [30]. Measurements of the weight and height were recorded and the BMI was calculated by the formula weight (kg)/height^2 ^(cm) [31].

Cardio-respiratory fitness was evaluated by a maximal walking test for estimation of peak oxygen uptake (VO_2peak_), according to the Balke modified protocol [32], using a multistage treadmill test of graded exercise. The estimated peak oxygen uptake (VO_2peak_) was computed from The American College of Sports Medicine (ACSM) formulas for graded walking (speeds ≤ 8 km/h, VO_2 _ml·kg^-1^·min^-1 ^= (0.1·ms^-1 ^+ 1.8·ms^-1^·inclination (%) + 3.5) or running (speeds > 8 km/h, VO_2 _ml·kg^-1^·min^-1 ^= (0.2·ms^-1 ^+ 0.9·ms^-1^·inclination (%) + 3.5) [33].

### Pulmonary function test

All participants underwent a pulmonary function test (PFT), evaluated by means of a spirometer (Spida 5, USB Spirometry from Micro Medical Ltd, Rochester, Kent, UK, 2006). The spirometric measurements were performed with the subject sitting upright with a nose clip attached. Spirometric testing was done by a trained physiotherapist in accordance with guidelines set by the American Thoracic Society and the European Respiratory Society (ATS/ERS) [34] and included measurements of:

FVC-Forced Vital Capacity: the maximal volume of air delivered during an expiration made as forcefully and completely as possible starting from full inspiration; that is, vital capacity performed with a maximally forced expiratory effort, expressed in liters.

FEV_1 _- Forced Expiratory Volume in one second: the volume, expressed in liters, delivered in the first second of the FVC manoeuvre.

PEF-Peak Expiratory Flow: the maximum expiratory flow achieved from a maximum forced expiration, starting without hesitation from the point of maximal lung inflation, expressed in liter/minute.

FEV_1_/FVC%-the absolute ratio; derived from observed values (not percent predicted) (liters/liters × 100). Primarily used in the diagnostics of obstructive ventilatory disease [35].

Additionally, observed values were expressed as percentage of predicted values to control for the influence of age, gender, weight and height. The published equations of the European Community for Coal and Steel (ECCS) [36] were internalized in the spirometry equipment and used as reference data. Additionally, data from the matched population controls served as references and basis for comparisons. Repeated measurements were performed until three acceptable manoeuvers were obtained, and the largest FVC and FEV_1 _values were recorded for further analysis. Based on these results, the patients were categorized as having a restrictive ventilatory pattern (FVC ≤ 80%, FEV_1_/FVC ≥ 70%, decreased or normal FEV_1_), obstructive ventilatory pattern (FEV_1_/FVC < 70%, decreased FEV_1_, normal or decreased FVC) or normal pulmonary function (FVC > 80%, FEV_1 _> 80%, FEV_1_/FVC > 80%) [37-39].

### Statistical analysis

Statistical analyses were performed using the SPSS software (Statistical packages for the social sciences) for Windows, version 17.0 (SPSS Norway, Oslo, Norway). The Independent sample t test was used for intergroup comparisons of continuous, normally distributed data and the Mann Whitney U test for comparisons of skewed distributions. Results are presented as mean (SD) or median (min-max) values. Intergroup comparisons of categorical data were analyzed using the Chi Square test. Results are presented as summaries of observed frequencies (counts) together with rounded percentages.

The associations among spinal mobility (BASMI) and disease activity (ASDAS) and pulmonary function (FVC%) are visualized with combined scatter and cumulative probability plots. These plots combine the FVC% score with the corresponding mobility (BASMI) and disease activity (ASDAS) score for each individual. The BASMI and ASDAS scores were plotted in cumulative order (from lowest value to highest). The combined procedure yielded a scatter plot (observation of two variables combined), in which one of the variables were plotted against its cumulative probability. Univariate ANCOVA analyses were performed to explore differences in pulmonary function between patients and controls adjusting for age, gender, height and smoking status. Further, a multiple linear model was used to estimate the explanatory power of independent variables (demographic, disease related, VO_2peak_) on restrictive pulmonary impairment (FVC%) [40,41]. A *P*-value of 0.05 was considered statistically significant.

## Results

### Participants

Two hundred and fifty patients with AS were invited, and 162 (59%) gave their informed consent to participate. Fifteen did not complete the pulmonary assessments, thus, a total of 147 (91%) of the 162 patients with informed consent were included in the analyses. Three hundred and twenty-nine letters were sent to invite potential controls and 139 (42%) accepted participation. Of these, a total of 121 of the 139 (87%) controls who accepted participation completed the pulmonary function tests and were included in the analyses.

For both the AS patients and the population controls, the participating subjects were older (*P *= 0.04 and *P *= 0.03, respectively) and a higher proportion were living in the western part of Oslo (*P *= 0.01, *P *= 0.06, respectively) compared with the subjects who rejected participation. There were no significant differences in gender between participating and non-participating subjects among AS patients and population controls.

### Demographic and disease related variables

Demographic variables of the AS patients and the population controls are shown in Table 1. Patients were younger (*P *= 0.01), more educated (*P *= 0.03) and a higher proportion were reported to receive social security benefits (*P *< 0.001) compared with controls. The other demographic parameters showed no significant differences between the groups.

**Table 1:** Characteristics of AS patients and population controls

Characteristics	AS Patients (*n *= 147)	Controls (*n *= 121)	*P *-value
Age (yr) median (range)	48.5 (30 to 70)	56.0 (30 to 70)	0.01^a^
Male n (%)	93 (63.3)	68 (56.2)	0.24^d^
Height (cm), mean (SD)	174.1 (9.7)	173.4 (8.9)	0.54^b^
Weight (kg) mean (SD)	77.4 (13.7)	77.5 (13.7)	0.98^b^
BMI (kg/cm^2^) mean (SD)	25.5 (3.5)	25.7 (3.7)	0.59^b^
ASDAS n (%)		-	
< 1.3 (low)	23 (15.6)		
1.3 to 2.1 (moderate)	45 (30.6)		
2.1 to 3.5 (high)	58 (39.5)		
> 3.5 (very high)	10 (10.9)		
BASFI	2.1 (0 to 10)	0.3 (0 to 6.3)	< 0.001^a^
BASMI	3.3 (1.8)	1.7 (0.9)	< 0.001^b^
CRP	3.0 (1.57)	1.0 (1.103)	< 0.001^a^
ESR	16.5 (1.90)	8.0 (1.70)	< 0.001^a^
VO_2peak _(ml/kg/min)	39.4 (8.0)	40.4 (7.5)	0.002^e^
Smoking			
Lifelong non-smoker, n (%)	74 (50.0)	59 (48.8)	0.81^c^
Ex-smoker, n (%)	48 (32.4)	38.2 (30.6)	
Current smoker, n (%)	26 (17.6)	22.9 (20.7)	
> 12 yr education, n (%)	103 (71)	68 (57)	0.03^d^
Currently employed, n (%)	111 (76.6)	99 (83.2)	0.18^d^
Social security benefit, n (%)	57 (39.4)	15 (12.6)	< 0.001^d^
Married/living with partner, n (%)	99 (67)	71 (60)	0.20^d^
Anti-TNF-alpha medication	32 (22)	-	

^a ^Mann Whitney U test, ^b^Independent Sample t-test, ^c^Chi Square test, ^d ^Chi Square test with correction for continuity (2 × 2 table), ^e^ANCOVA adjusted for age and education.

The inflammatory marker values were significantly higher in patients than population controls (CRP, *P *< 0.001, ESR, *P *< 0.001). Furthermore, the population controls had significantly better aerobic capacity (VO_2peak_, *P *= 0.002), better self-reported physical function (BASFI, *P *< 0.001) and less restricted spinal mobility (BASMI, *P *< 0.001). Half of the AS patient group had high or very high disease activity (ASDAS ≥ 2.1) (Table 1).

### Pulmonary function in patients and controls

The PFT showed significantly lower values for AS patients compared to controls with regard to FVC% (97 vs 105, *P *< 0.001), FEV_1_% (90 vs 99, *P *< 0.001) and PEF% (95 vs 99, *P *= 0.05). Most of the AS patients were categorized with normal pulmonary function, but 18% (*n *= 27) were categorized with restrictive pattern. As none of the population controls showed a restrictive pattern, the proportion of pulmonary impairment in the two groups was significantly different (*P *< 0.001) (Table 2). Approximately 10% of the AS patients and 9% of the controls were categorized with obstructive pattern.

**Table 2:** Measures of pulmonary function in AS patients and controls

PFT	AS patients (*n *= 147)	Controls (*n *= 121)	ß (95% CI)	*P*-value
FVC (liters) mean (SD)	4.0 (1.2)	4.1 (1.0)	0.3 (0.14,0.45)	< 0.001^a^
FVC%	97.2 (18.1)	104.9 (15.2)		< 0.001^b^
FEV_1 _(liters) mean (SD)	3.1 (0.9)	3.2 (0.9)	0.3 (0.18,0.43)	< 0.001^a^
FEV_1_%	89.8 (16.0)	98.5 (14.5)		< 0.001^b^
PEF (liters/minute) mean (SD	464.7 (121.4)	469.1 (120.1)	20.5 (1,41.1)	0.05^a^
PEF%	95.2 (17.6)	99.4 (16.6)		0.05^b^
FEV_1_/FVC%	76.5 (7.5)	77 (6.4)		0.38^b^

*Respiratory pattern*				
Normal n (%)	105 (71.4)	110 (90.9)		< 0.001^c^
Restrictive pattern n (%)	27 (18.4)	0		< 0.001^c^
Obstructive pattern n (%)	15 (10.2)	11 (9.1)		0.76^c^

FVC, forced vital capacity; FEV_1_, forced expiratory volume in one second; PEF, peak expiratory flow; FVC%, FEV_1_%, PEF%, predicted percentages adjusted for age, gender, smoking and height (adjustments made in the device); FEV_1_/FVC%, absolute ratio (derived from observed values). A, linear regression, adjusted for age, gender, smoking and height; b, t-test; c, chi-square test

Among the 27 AS patients with a restrictive pattern, 21 (78%) were males, median age was 57 years (range 33, 68), median disease duration was 27 years (range 18, 46), 9 (33% were using biological anti-tumor necrosis factor therapy and 15 (56%) were lifelong non-smokers. Patients with restrictive pattern were significantly older (*P *= 0.015) and had longer disease duration (*P *= 0.054) than patients with normal pulmonary function (Table 3). Furthermore, patients with restrictive pattern had significantly reduced spinal mobility (BASMI, (*P *< 0.001), chest expansion (*P *< 0.001), lumbar flexion (*P *< 0.001) and lateral lumbar flexion (*P *< 0.001) compared to patients with normal pulmonary function. The occurrence of increased dorsal kyphosis (measured as TWD) was significantly higher in patients with restrictive ventilatory pattern with a median of 18.5 cm (range 11, 38) compared to patients with normal pulmonary function median 12 cm (range 8, 44, *P *< 0.001). Additionally, physical function (BASFI, *P *= 0.003) and cardio-respiratory fitness (VO_2peak_, *P *= 0.002) were significantly reduced in patients with restrictive respiratory impairment.

**Table 3:** AS patients with normal pulmonary function versus patients with restrictive pattern

	Normal pulmonary function (*n *= 105)	Restrictive pattern (*n *= 27)	*P*-value
Age (yr), median (min, max)	46 (30,70)	57 (33,68)	0.015
Males, n (%)	66 (63)	21 (78)	0.218
Disease duration (yr)	22 (7,55)	27 (8,46)	0.054
Lifelong non-smokers, n (%)	53 (51)	15 (56)	0.799
FVC (liters), mean (SD)	4.3 (1.1)	2.8 (0.7)	< 0.001
FVC %	103.4 (12.8)	69.4 (6.6)	< 0.001
FEV1 (liters)	3.4 (0.9)	2.2 (0.5)	< 0.001
FEV1 %	96.6 (12.2)	68.6 (7.9)	< 0.001
PEF (liters/minute)	493.2 (119.9)	400.0 (101.3)	< 0.001
PEF %	100.0 (16.0)	85.5 (15.8)	< 0.001
FEV1/FVC %	77.4 (6.1)	79.4 (8.3)	0.158
ASDAS	2.8 (1.0)	3.1 (0.9)	0.140
BASDAI	4.1 (2.1)	4.3 (2.0)	0.709
BASFI	1.8 (0.8, 8.0)	2.9 (0.9, 10.0)	0.003
BASMI	2.9 (1.6)	5.4 (1.8)	< 0.001
Chest expansion (cm)	4.6 (2.1)	2.9 (1.8)	< 0.001
Lumbar flexion (cm)	4.3 (1.4)	2.4 (1.6)	< 0.001
Lumbar lateral flexion (cm)	14.4 (5.6)	7.6 (5.5)	< 0.001
TWD (cm)	11.8 (7.8, 43.5)	18.5 (10.5, 38.0)	< 0.001
CRP	3.0 (1.0, 57.0)	7.0 (1.0, 28.0)	0.063
ESR	15.0 (2.0, 83.0)	28.0 (1.0, 90.0)	0.006
VO_2_peak	41.0 (7.2)	35.9 (8.9)	0.002
Anti-TNF-α-medication, n (%)	21 (20)	9 (33)	0.201

PFT results, clinical and laboratory measures in the AS patients (*n *= 132).

Mann Whitney U test for comparisons of skewed distributions, Independent Sample T-test for normally distributed data. FEV1%, FVC% and PEF% are adjusted for age, gender and actual height. FEV1/FVC % = absolute FEV1/FVC ratio. The other measurements are not adjusted. Chi-Square test for comparisons of categorical data

### Smoking

When comparing AS patients who never smoked (*n *= 74) with ex-smokers/current smokers (*n *= 73), we found statistically significant differences (ex-smokers/smokers worse health) in all measures of disease activity (ASDAS, BASDAI, ESR, CRP) (Table 4). Additionally, chest expansion and VO_2peak _were significantly poorer in the group of smokers and ex-smokers. However, there were no significant differences in pulmonary function, self-reported pulmonary disease or physical function between AS patients who never smoked and ex-smokers/current smokers. The group of smokers/ex-smokers was less frequently on biologic treatment (*P *> 0.007).

**Table 4:** Comparisons of PF and clinical variables with regard to smoking habits in AS patients

Test	Lifelong non-smokers(*n *= 74)	Ex-smokers + current smokers (*n *= 73)	*P*-value
Age^a ^(yr)	47.8 (11.8)	51.1 (10.1)	0.07^c^
Disease duration^b ^(yr)	21.5 (7 to 55)	23 (8 to 47)	0.64^d^
Self reported pulmonary disease (yes/no) n (%)	6 (8.1)	10 (13.9)	0.53^e^
FEV_1 _(liters)^a^	3.2 (1.0)	3.0 (0.9)	0.18^c^
FEV_1 _%^a^	89.9 (15.7)	89.6 (16.5)	0.90^c^
FVC (liters)^a^	4.1 (1.2)	3.9 (1.1)	0.45^c^
FVC %^a^	95.7 (17.4)	98.6 (18.8)	0.33^c^
PEF (liters/minute)^a^	469.3 (128.5)	458.8 (114.7)	0.60^c^
PEF %^a^	93.6 (18.0)	96.7 (17.1)	0.28^c^
FEV_1_/FVC %^a^	77.5 (7.4)	75.4 (7.6)	0.09^c^
ASDAS	2.6 (1.0)	3.0 (0.9)	0.004^c^
BASDAI^a^	3.8 (2.0)	4.5 (2.0)	0.040^c^
ESR^b^	12 (1 to 90)	19 (2 to 67)	0.047^d^
CRP^b^	3 (1 to 57)	5 (1 to 52)	0.018^d^
BASFI^b^	1.8 (0 to 10)	2.3 (0 to 8.5)	0.66^d^
BASMI^a^	3.3 (1.8)	3.2 (1.8)	0.74
Chest expansion (cm)^a^	4.8 (2.3)	3.9 (2.0)	0.019^c^
Lumbar flexion (l-Schober)^a^	4.0 (1.7)	4.0 (1.7)	0.90^c^
Lumbar lateral flexion^a^	12.9 (6.0)	13.6 (6.3)	0.53^c^
Tragus to wall distance^b^	13.1 (7.8 to 38.0)	12.0 (8.0 to 43.5)	0.33^d^
VO2max^a^	40.9 (8.4)	38.0 (7.4)	0.037^c^
Anti-TNF-α-med, n (%)	23 (31)	9 (13)	0.007
*Respiratory pattern*			
Normal (n) (%)	53 (71.6)	52 (71.2)	1.00^e^
Restrictive pattern (n) (%)	15 (20.3)	12 (16.4)	0.67^e^
Obstructive pattern (n) (%)	6 (8.1)	9 (12.3)	0.43^e^

^a ^Mean (SD). ^b ^Median (min-max). ^c ^Independent Sample T test. ^d ^Mann Whitney U test. ^e ^Chi Square test

### Associations between pulmonary function and relevant variables

**Figure 1:**
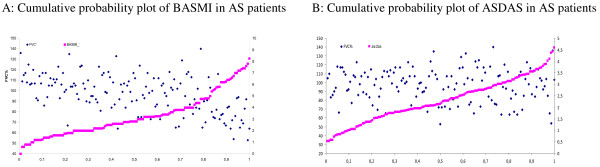
**Cumulative probability plots of spinal mobility (BASMI) and disease activity (ASDAS) versus FVC%**. The probability plot of the BASMI scores (squared symbols) visualizes that patients with high BASMI scores (the curve bends steeply at approximately 20%) almost always had low FVC%, while most of the patients with low BASMI scores also had normal FVC%. The B plot shows that among the patients with very high ASDAS scores (approximately 15%), most had normal FVC%.

Figure 1 presents scatter plots of the spinal mobility scores (BASMI) and the disease activity scores (ASDAS) in cumulative order versus the FVC% score. The information obtained from these plots can be exemplified by the association between BASMI and FVC% (Figure 1A): The probability plot of the BASMI scores visualizes that approximately 20% of the patients had a BASMI score of 4.5 or more. Almost all these patients had a FVC% score less than 80 (indicating restrictive respiratory impairment), whereas for disease activity (ASDAS), this association is lacking (Figure 1B).

A multiple regression model was built to assess how demographic and clinical variables and aerobic capacity could predict the variation in FVC% in the patient group. Male gender, BASMI and chest expansion contributed independently and significantly, and the final model explained 45% of the variance in FVC% (*P *< 0.001) (Table 5).

**Table 5:** A multiple linear regression model for 147 AS patients, dependent variable FVC%

	Crude Estimates ß^a ^(95% CI^b^)	*P*-value	Adjusted estimates^c ^ß (95% CI)	*P*-value	Final model (R^2^)
*Age*	-0.3 (-0.6, -0.05)	0.02	ns		0.45
*Gender*					*P *< 0.001
Female	Reference	< 0.01	-8.2 (-12.9, -3.5)	0.001	
Male	-8.3 (-14.2, -2.3)				
*Smoking*^d^					
Lifelong non-smokers,	Reference	0.33	ns		
Ex-smokers and smokers	2.9 (-3.0, 8.8)				
*Education*^b^					
≤ 12 years	Reference	0.94	ns		
> 12 years	0.3 (-6.3, 6.8)				
*ASDAS*	-1.4 (-4.5, 1.7)	0.36	ns		
*BASFI *(0 to 10)	-1.9 (-3.3, -0.5)	< 0.01	ns		
*BASMI *(0 to 10)	-5.6 (-6.9, -4.3)	< 0.001	-4.1 (-5.4, -2.7)	< 0.001	
*Chest expansion (cm)*	4.0 (2.8, 5.1)	< 0.001	2.7 (1.6, 3.9)	< 0.001	
*VO_2_peak*	0.6 (0.2, 0.9)	< 0.001	ns		

^a ^Estimated regression coefficients, ^b ^confidence interval, ^c ^Adjusted for the other variables in the table, ^d ^Smoking (lifelong non-smokers vs ex-smokers and smokers). ^e ^Education (≤ 12 years or > 12 years), ns: not significant in the adjusted model

## Discussion

This study showed significantly impaired pulmonary function in the AS patients compared to reference data and to the population controls. Furthermore, significant associations were found between pulmonary function and the typical clinical features of AS: reduced spinal-and chest-wall mobility.

The results of this study are in agreement with previous studies, although a lower prevalence of restrictive abnormalities was observed. We found a prevalence of 18% of restrictive disorders, compared to the reported prevalence between 20 and 57% in other studies [4,8,12,14,15,18,20,21,42,43]. However, these studies were based on small sample sizes, ranging from 17 to 55 subjects, potentially influencing the representativeness. Hence, the results of this study indicate that restrictive pulmonary function may be a consequence of AS, but the prevalence of restrictive involvement may be lower than previously reported.

Another possible explanation of the lower prevalence of restrictive impairment found in this study may be attributed to the assessment of pulmonary function. Lung volumes are related to body size, and standing height is the most important correlating variable [39]. However, patients with AS often loose height due to increasing dorsal kyphosis as the disease progresses. Some of the previous studies have used patient's original height or height from arm span measurement to calculate predicted normal values [6,44]. Yet, since the reliability of recalled height may be questionable [39], the actual height measured at time of testing was used in this study. Hence, for patients who have lost height, the comparisons of PFT results were done with reference values appropriate for originally shorter persons. As a consequence, we may have failed to discover some cases of restrictive pulmonary impairment and, consequently, underestimated the prevalence.

Furthermore, the population in this study was well educated, married or living with a partner, primarily of Norwegian ethnicity and recruited from a non-industrial (sub-) urban district area with high socio-economic status (SES). Within the city of Oslo, differences in health, exercise habits and mortality rates between districts are significant, and strongly related to SES [45]. Adverse effects of low SES on pulmonary function are well documented, as low SES often is associated with unfavorable environmental conditions, such as increased exposure to indoor and outdoor pollution, increased occupational exposures and decreased access to health care. Moreover, genetic factors influencing lung function may be attributable to differences in SES [37]. The high SES values in this sample may be associated with the low prevalence of pulmonary impairment.

Reduced spinal mobility and chest expansion and male gender made the largest contributions to explaining the variance of pulmonary function in the patient group. This result is in accordance with several previous studies, reporting that the restrictive pulmonary disorder seen in AS patients is associated with increased stiffness and ankylosis of the spine and costovertebral joints [9,10,46]. However, neither measures of acute inflammation, disease activity, smoking, physical function nor cardio-respiratory fitness contributed to explaining pulmonary function in this study. The results indicate that inflammatory activity is of less importance with regard to restrictive pulmonary function in this patient group, but a causal inference cannot be drawn due to the cross sectional design. The findings do, however, support the assumption of an association between musculoskeletal limitations and restrictive pulmonary impairment, underlining the importance of maintained spinal flexibility in the management of AS.

Earlier reports have differed concerning whether disease duration is associated with restrictive pulmonary impairment in AS or not. This is interesting, because AS is a chronic, progressive disease. If pulmonary restrictivity is related to musculoskeletal limitations progressing with time, a logical consequence would be a parallel deterioration in pulmonary parameters. In this study, patients with restrictive pattern were, in agreement with these expectations, significantly older and had longer disease duration than patients with normal pulmonary function.

Smoking is recognized as having a negative impact on patients with respiratory restrictions, independent of the etiology of the restriction [20]. Yet, in this study, when comparing lifelong non-smokers (*n *= 74) with ex-smokers and current smokers (*n *= 73), we found no significant differences in pulmonary function, self-reported pulmonary disease or physical function between the AS patients. This result is in agreement with previous reports [6,7,13]. Surprisingly, we found significant differences between these two groups concerning all measures of acute inflammation and disease activity, indicating that smoking is connected to the general inflammatory process in AS. Earlier reports on pulmonary function in AS found no differences in disease activity between smokers and non-smokers [4,20]. However, there are previous descriptions of an association between smoking and a more rapid disease progression and a poorer long-term outcome of AS [47,48]. The findings of the present study indicate that smoking is connected to measures of disease activity, chest expansability and cardio-respiratory fitness, but probably not directly to measures of pulmonary function in AS. Furthermore, similar observations were recently made in an early axial spondyloarthritis cohort: Patients who smoked were more likely than non-smokers with the disease to have higher disease activity, poorer functional status, increased axial inflammation and increased axial structural damage on MRI [49]. Moreover, according to the results of the current study, it seems that smokers are less frequently treated with biological agents.

The gold-standard definition of restrictive pulmonary disease requires measurement of total lung capacity (TLC). Spirometry is very effective at excluding a restrictive defect, but a classic restrictive pattern on spirometry does not accurately predict a true restrictive defect because it represents a true restriction in less than 60% of cases (38). Thus, measurement of lung volumes (TLC) is necessary to confirm a restrictive impairment (39). As data from TLC measurements were not available in this study, the true prevalence of restrictive impairment may be even smaller than reported, and lung volume data would have provided a more precise estimate.

We did not produce a category for mixed ventilatory abnormalities in this study. A mixed defect is characterized by the co-existence of obstruction and restriction (39), defined by reduced TLC and reduced FEV_1_/FVC ratio. There were four cases of possible mixed abnormalities among the AS patients in the present sample; all had FVC scores well below 80% of predicted (indicating restriction) combined with a FEV_1_/FVC ratio just below 0.7 (indicating obstruction). Because FVC may be equally reduced in both obstruction and restriction, we evaluated the individual flow-volume-curves (which appear differently for restrictive and obstructive defects [39]) before categorization, and we let the FVC% score overrule the ratio. All four cases of doubt were categorized with restrictive ventilatory pattern.

Another weakness of this study is the lack of data on radiological changes in skeletal structures. Previous reports have recognized relationships between radiographic manifestations and BASMI (especially lumbar flexion and lumbar lateral flexion), but yet, spinal mobility measures cannot stand proxy for radiographic evaluation in an individual patient [50]. An additional weakness is the lack of CT imaging, as interstitial lung disease is diagnosed by CT. However, there is little evidence of correlation between lung findings by imaging and abnormalities measured by spirometry [11,12,14,16,21]. This study is, however, strengthened by the relatively large number of subjects included, providing an opportunity to produce more accurate estimates and, hopefully, a more representative sample. Furthermore, the comparisons with controls randomly drawn from the general population and a comprehensive clinical examination may also strengthen the results.

## Conclusion

This study showed that patients with AS were more likely to have restrictive respiratory impairment compared to controls and reference data. However, the prevalence of respiratory impairment in this study was lower than previously reported. The reduced pulmonary function was closely related to reduced spinal- and chest wall mobility, whereas measures of disease activity, physical function, smoking and cardio-respiratory fitness did not contribute significantly in explaining pulmonary function. The results emphasize the importance of maintaining spinal flexibility in the management of AS. However, the effects of mobility and aerobic exercise on pulmonary function in AS patients remain to be explored. The study exposed a need for further examination of the relationships between the disease specific changes and pulmonary function in AS. Furthermore, patients with severely reduced spinal mobility should be referred to pulmonary function examination and relevant follow-up treatment.

## Abbreviations

AS: ankylosing spondylitis; ASDAS: Ankylosing Spondylitis Disease Activity Score; BASFI: Bath Ankylosing Spondylitis Functional Index; BASMI: Bath Ankylosing Spondylitis Metrology Index; BMI: body mass index; CRP: C-reactive protein (mg/l); FEV_1_: forced expiratory volume in one second (liters); FEV_1_/FVC% (liters/liters × 100); FVC: forced vital capacity (liters); FVC%: percentage of predicted controlled for the influence of confounding factors; PEF: peak expiratory flow (liter/minute); PF: pulmonary function; PFT: Pulmonary Function Test; TLC: total lung capacity; ESR: erythrocyte sedimentation rate (mm/hr); VO_2peak_: peak oxygen uptake.

## Competing interests

The authors declare that they have no competing interests.

## Authors' contributions

GB performed the statistical analysis, interpreted the data, and drafted, wrote and revised the manuscript. SH participated in the data collection process and in statistical analysis and interpretation of data. DVDH and MM contributed to analysis and interpretation of data and were involved in revising the manuscript critically for important intellectual content. HD was responsible for the conception and design of the study, the acquisition of data, statistical analysis, and drafting and revising the manuscript. All authors read and approved the final manuscript.

## Authors' information

This group of authors has extensive experience and qualifications in the fields of physiotherapy, internal medicine and rheumatology. GB and SH are PTs and research fellows. DVDH is an MD, professor in rheumatology at Leiden University Medical Center, Leiden, The Netherlands. MM is an MD, head of the department of General Internal Medicine, Oslo University Hospital and associated professor on the Faculty of Medicine, University of Oslo, Norway. HD is a PT, PhD and senior researcher at the National Resource Center for Rehabilitation in Rheumatology, Department of Rheumatology, Diakonhjemmet Hospital, which has status as EULAR Centre of Excellence in rheumatologic research in Europe (http://www.eular.org).
